# Non-Invasive Contactless Tracking of Respiratory Rate and Heart Rate During Sleep

**DOI:** 10.3390/s26041082

**Published:** 2026-02-07

**Authors:** Susana Mejía, Isabel Cristina Muñoz, Fabián Andrés Castaño, Alher Mauricio Hernández

**Affiliations:** Bioinstrumentation and Clinical Engineering Research Group—GIBIC, Bioengineering Department, Engineering Faculty, Universidad de Antioquia UdeA, Calle 70 No. 52-21, Medellín 050010, Colombia; susana.mejiae@udea.edu.co (S.M.); isabelc.munoz@udea.edu.co (I.C.M.); fabian.castano@udea.edu.co (F.A.C.)

**Keywords:** Smart Bedding, respiratory rate, heart rate, polysomnography, signal processing, sleep tracking

## Abstract

Heart and respiratory rate monitoring during sleep enables the detection of physiological irregularities through contact or contactless methods. Traditional approaches like polysomnography are accurate but costly, ergonomically limited, and often poorly accepted by patients. Smart Bedding^®^ is a novel, flexible bedsheet equipped with a high-resolution sensor network that records movement, pressure, sound, temperature, and humidity throughout the night. This study aimed to estimate cardiorespiratory parameters using the Smart Bedding^®^ IMU. Data from 30 participants sleeping on Smart Bedding^®^ while undergoing simultaneous polysomnography were analyzed. A robust and low-cost preprocessing pipeline was developed; estimation was performed using zero-crossing, peak detection, and Burg’s method for comparison, and validation was conducted using polysomnography as the gold-standard reference. Respiratory and heart rates were accurately estimated, achieving overall accuracies of 93.9% and 88.7% using zero-crossing and peak detection, respectively. Respiratory rate estimation showed no significant limitations across the frequency spectrum or among sleeping positions. However, heart rate estimation accuracy decreased when the frequency was below 55 BPM or when participants slept in a lateral sleep position, likely due to reduced cardiac signal power. Overall, the proposed methodology accurately tracked respiratory and cardiac patterns throughout the night, supporting Smart Bedding^®^ as a promising tool for future sleep tracking applications.

## 1. Introduction

Monitoring respiratory rate (RR) and heart rate (HR) during sleep is essential for assessing sleep quality and various sleep disorders [1]. Accurate tracking of these vital signs can reveal irregularities that indicate conditions such as sleep apnea, which significantly impacts cardiovascular health [2]. Persistent disruptions in breathing patterns and heart rhythms during sleep can lead to hypertension, heart disease, and stroke [3]. Furthermore, some findings [4,5] indicate that poor sleep is a risk factor for cognitive decline such as dementia and Alzheimer’s disease.

Measuring RR and HR can be accomplished through contact or contactless methods. Polysomnography (PSG) is the gold-standard contact method for measuring physiologic variables during the night using sensors and surface electrodes for the continuous recording of electroencephalogram, electro-oculogram, electromyogram, electrocardiogram, nasal airflow, and respiratory effort [6]. The respiratory and cardiac signals can be used to estimate RR and HR, respectively. Other contact techniques widely used to record HR and RR are photoplethysmography [7], pulse oximetry, and advanced cardiorespiratory monitoring systems. Contact methods are the most common for measuring these physiological aspects because of their high accuracy [8]. However, they are often associated with high costs, suboptimal ergonomics, poor patient acceptance [9], and they are impractical for long-term sleep monitoring and limited only to clinical usage.

New contactless systems [8,10,11,12,13,14,15,16,17] have been proposed in both industry and academia to avoid patient discomfort and promote home-based measurements. Most of these technologies estimate vital signs using alternative data sources, such as wireless signals [10], ballistic force [13], non-contact radar signals [14], ultrasound [15], audio signals [16], and pressure [17]. Despite their advantages, some of these methodologies report difficulties in installation (e.g., specific requirements for the bed or mattress and requiring the same sleeping position) and challenges in measurement when there is movement during sleep [18]. Additionally, due to the low power of physiological signals, there is a dependency on the wave propagation capacity over the mattress and bed to the inertial sensors [13], which has led some researchers to position the sensors on the chest [19,20] or hand, like smartwatches [21] or rings [22], potentially causing discomfort during sleep. A further challenge for these technologies is integrating the estimation techniques directly into microprocessors to minimize cloud processing, reduce costs, and provide efficient real-time analysis.

This article focuses on the estimation of RR and HR using Smart Bedding^®^ (SB), a novel flexible bedsheet. Both estimations were performed using time and frequency techniques, such as peak detection, zero-crossing, and Burg’s method. The aim was to verify which approach is accurate, robust, low-cost, and easy to use in the microprocessor. The validation was conducted using data collected from a database containing thirty healthy subjects sleeping on Smart Bedding^®^ while undergoing simultaneous PSG monitoring.

## 2. Smart Bedding^®^ Overview

Smart Bedding^®^ (SB) is a flexible device developed by the Bioinstrumentation and Clinical Engineering Research Group (GIBIC, Medellín, Colombia). It is positioned beneath the conventional bedsheet, enabling users to rest on it without direct contact.

**Figure 1:**
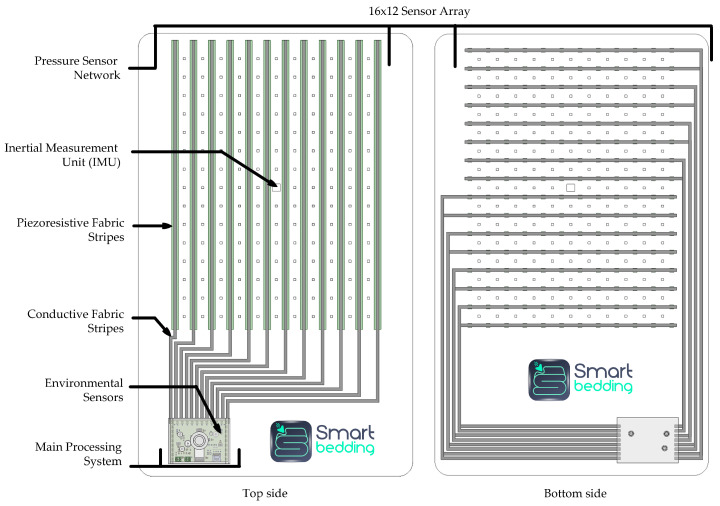
Smart Bedding^®^ hardware schematic.

As shown in Figure 1, the SB bedsheet incorporates a high-resolution sensor network that measures, comfortably and non-invasively, four variables selected for their relevance in sleep analysis. To assess the pressure exerted by the body on the mattress, the bedsheet includes a 16 × 12 array of sensors strategically arranged so that the user’s chest lies over them, enabling the detection of body positions [23]. Movement is recorded by an IMU located at the center of the bedsheet, which integrates a three-axis accelerometer and a three-axis gyroscope. Sound is captured through a microphone, while environmental temperature and humidity are measured by dedicated sensors.

## 3. Materials and Methods

### 3.1. Data Acquisition

Thirty healthy subjects, 13 men and 17 women (age 32.7 ± 8.9, weight 67.6 ± 15.1 kg, height 1.67 ± 0.1 m, and BMI 24.07 ± 3.4 kg/m^2^), were selected for data collection after verifying inclusion and exclusion criteria (ages between 18 and 65 years old; not pregnant; no alcohol, drugs, or hallucinogens consumed in the last 48 h; not under medical treatment affecting brain activity; no recent head or thoracic injuries or traumas; no neurological or psychiatric disorders such as epilepsy, Parkinson’s disease, or schizophrenia; no pacemakers or implanted electrical stimulators; and no physical disabilities that impede movement or getting out of bed). The study was conducted under an experimental design approved by the Ethics Committee of the University of Antioquia (approval report No. 420), with all participants providing written consent.

All subjects underwent a full-night PSG sleep over SB strategically placed between the mattress and the bed’s sheet. PSG recordings include electroencephalographic, electromyographic, electro-oculographic, and electrocardiographic signals, as well as thoracic effort, respiratory airflow, and oxygen saturation. In addition, continuous video and audio recordings are acquired throughout the night. All data were acquired using the Natus^®^ Quantum (Natus, Middleton, WI, USA) with a sampling rate of 1024 Hz (software NeuroWorks 3.4), and all electrodes and sensors were placed on the volunteers according to the American Academy of Sleep Medicine (AASM) guideline. SB records motion data at a sampling rate of 20 Hz using gyroscope sensors. Due to the research objectives, the signals derived from PSG and SB were synchronized. All data were processed using MATLAB R2023b.

### 3.2. Respiratory and Heart Rate Estimation

**Figure 2:**
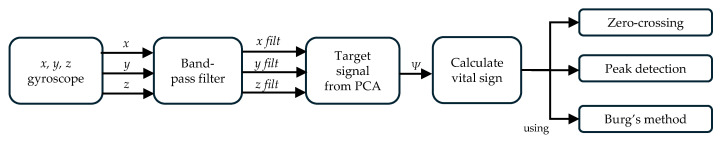
Pipeline diagram for respiratory and heart rate estimation using gyroscope signals and three different methods for evaluation: zero-crossing, peak detection, and Burg’s method.

Three different methods were used to detect heart and respiratory rate from SB motion data: zero-crossing, peak detection, and Burg’s method. These techniques were selected due to their widespread and well-known use in this field, as well as their ease of implementation on embedded microprocessors. Due to the nature of the gyroscope data and their susceptibility to being significantly affected by a person’s movements during sleep, raw signals were not utilized for detecting vital signs. Instead, the methodology shown in Figure 2 was used to enhance the target signal from which RR and HR were extracted.

Both HR and RR were monitored every minute for each volunteer. Within each one-minute epoch, the quantity of movement (*G*) was calculated using Equation (1) from the *x*, *y*, and *z* gyroscope signals [24] and normalized by the epoch length *L*. To avoid inaccurate estimations, the HR and RR from epochs with high movement (*G* > 0.7°/s) were set to the previous epoch’s measurement.

(1) $$G=\frac{\sum \sqrt{{\left(x_{i+1}-x_{i}\right)}^{2}+{\left(y_{i+1}-y_{i}\right)}^{2}+{\left(z_{i+1}-z_{i}\right)}^{2}}}{L}$$

**Figure 3:**
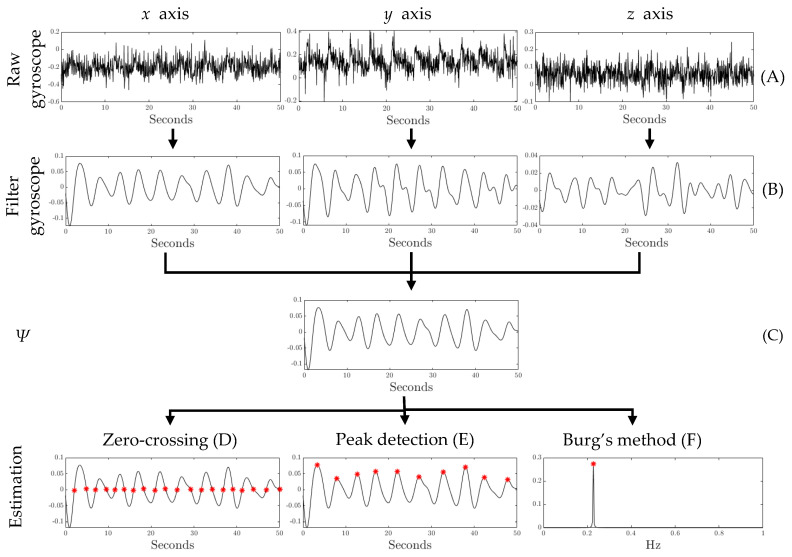
Step-by-step signals for respiratory rate estimation: (**A**) raw 3D gyroscope signals for one selected epoch, (**B**) Butterworth band-pass filter for all signals, (**C**) target signal after PCA implementation, and estimation using (**D**) zero-crossing, (**E**) peak detection, and (**F**) Burg’s method. Red asterisks denote the zero-crossings, detected peaks, and value from the Burg’s method.

In both cases, a Butterworth band-pass filter was employed on *x*, *y*, and *z* signals to extract only the relevant frequencies (Figure 3B), such as those between 0.16 and 0.4 Hz (equivalent to 9.6 and 24 BrPM) for RR and those between 0.7 and 2 Hz (equivalent to 42 and 120 BPM) for HR. Due to the arbitrary position of the person on the SB sheet and the constant changes in posture through the night, it is necessary to identify which of the three filtered signals contains the most information. For this purpose, a classical Principal Component Analysis (PCA) was performed, as shown in Figure 3C. The calculated eigenvalues ($\lambda_{1},\lambda_{2},\lambda_{3}$) were normalized according to Equation (2). The final target signal (Ψ) is defined by Equation (3) based on the resulting weights [19].

(2) $$\eta_{i}=\frac{\lambda_{i}}{\lambda_{1}+\lambda_{2}+\lambda_{3}};\;\;i=1,2,3$$

(3) $$\Psi =\eta_{1}xfilt+\eta_{2}yfilt+\eta_{3}zfilt$$

Finally, three different methods were used to estimate HR and RR from the target signal (Ψ): two in the time domain (Figure 3D,E) and one in the frequency domain (Figure 3F). Table 1 describes the detection procedure and the parameters used for both vital signs. In several cases, the signals exhibit small fluctuations that do not correspond to a heartbeat or respiration. To avoid estimation errors, restrictions for zero-crossings and peak detection were established.

### 3.3. Validation

The RR and HR real values were obtained from thoracic effort and ECG signals, respectively, derived from each subject’s PSG, excluding epochs where movement or position transitions were detected. To determine the precision of the estimation, the results were compared across different cardiac and respiratory frequency bands and sleeping positions, thereby accounting for postural effects on signal quality inherent to the contactless sensing configuration. The ground truth sleeping positions were labeled using overnight PSG video recordings.

The comparison was performed for each individual’s epoch-by-epoch estimation using two metrics: difference in the number of breaths or beats between the real and predicted values (Equation (4)) and accuracy (Equation (5)), where $y_{i}$ is the real value obtained from PSG signals and ${\dot{y}}_{i}$ is the predicted value for epoch i using the proposed method.

(4) $$Diff_{i}=\left|y_{i}-{\dot{y}}_{i}\right|$$

(5) $$Accuracy_{i}=\left(1-\frac{Diff_{i}}{y_{i}}\right)\times 100\left(\%\right).$$

In addition, Bland–Altman analysis was employed to assess the agreement between the proposed methods and the real cardiorespiratory values.

## 4. Results

To evaluate the accuracy of the real and predicted data using time and frequency techniques, the SB sheet was placed on the volunteer’s bed, and a PSG was conducted simultaneously. All processing algorithms were applied for each subject’s data from sleep onset to the end of sleep (8.1 ± 0.6 h), equivalent to 14,188 one-minute epochs in total, of which 804 were not calculated but rather carried over from the previous epoch due to a *G* value greater than 0.7°/s. These represent 5.6% of the total recorded epochs.

As performance indicators, execution time and memory usage on the microprocessor were evaluated to identify the estimation method that imposes the highest computational burden for continuous overnight monitoring of approximately 8 h. For a one-minute epoch, the preprocessing stage from raw gyroscope signals to the construction of the signal Ψ (Equation (3)) required on average 47,150 µs for RR estimation and 5962 µs for HR estimation, with the difference mainly attributed to the type of filtering applied in each case. The estimation step required 566 µs, 569 µs, and 10,290 µs when using zero-crossing, peak detection, and Burg’s methods, respectively. In addition, the memory consumption was similar for zero-crossing and peak detection (8.34 kB and 8.37 kB, respectively), while it was 24.78 kB for the Burg’s method. These results demonstrate that, despite its advanced spectral formulation, Burg’s method is considerably more computationally demanding, making it less suitable for resource-constrained microcontrollers operating in continuous overnight monitoring scenarios.

### 4.1. Respiratory Rate Estimation

**Figure 4:**
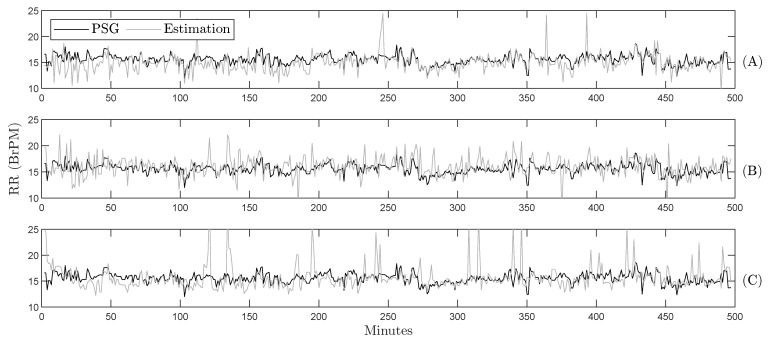
Gold standard and estimated RR during the whole night for one volunteer using the proposed methodology and different estimation techniques: (**A**) zero-crossing, (**B**) peak detection, and (**C**) Burg’s method.

Table 2 summarizes the experimental results for RR prediction using all time and frequency techniques. Regardless of the technique used, the third quartile of the data exhibits over 90.0% accuracy, showing consistent performance across sleeping positions and respiratory frequency ranges during the volunteers’ nights. However, zero-crossing detection yields a median difference of 0.8 BrPM for RR values below 17.4 BrPM and is the method that best predicts breathing regardless of the position over SB. The analysis indicates no correlation between prediction errors and sleeping position or low-movement periods, also evidenced in Figure 4, which illustrates the respiratory pattern throughout the night. The zero-crossing method (Figure 4A) most accurately reproduces the respiratory dynamics in agreement with the sleep patterns identified in the PSG.

**Figure 5:**
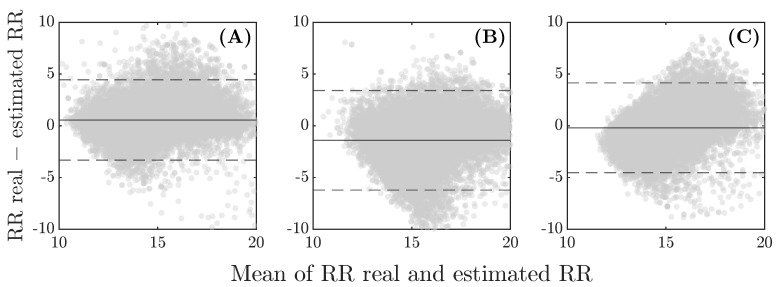
Bland–Altman plots for RR validation using (**A**) zero-crossing, (**B**) peak detection, and (**C**) Burg’s method. Dashed lines represent the limits of agreement (mean difference ± 1.96 standard deviations), and solid lines represent the mean difference between the predicted and measured values.

A between-subject analysis was conducted to validate the overall performance of each method. In general, without distinguishing between frequency ranges or sleeping positions, the three methods demonstrate good estimation performance, with median differences of 0.9, 1.6, and 1.3 BrPM and accuracies of 93.9%, 90.4%, and 92.1% for the zero-crossing, peak detection, and Burg methods, respectively. The Bland–Altman results (Figure 5) suggest no systematic discrepancy between the actual and predicted RR, as most data points are concentrated near the zero-difference line, and more than 94% fall within the limits of agreement. Notably, the plots exhibit low offsets: 0.6 BrPM for the zero-crossing technique (Figure 5A), −1.4 BrPM for peak detection (Figure 5B), and −0.2 BrPM for Burg’s method (Figure 5C).

### 4.2. Heart Rate Estimation

Table 3 describes the differences in and accuracy of HR estimation for 30 subjects. Results show that all estimation methods achieve good predictive performance when the nocturnal HR ranges between 56 and 70 BPM—a range observed in 50% of the volunteers—while all methods were sensitive to very low frequencies. Moreover, the peak detection technique stands out at a higher HR (more than 71 BPM), achieving a median accuracy of 95.6%. Although the overall adjustments are slightly lower compared to those reported for RR detection (Table 2), the methodology demonstrates a good level of consistency, with median accuracies above 84% across all positions on the bedsheet. However, prediction performance decreases in the lateral position, as evidenced by the high interquartile range.

**Figure 6:**
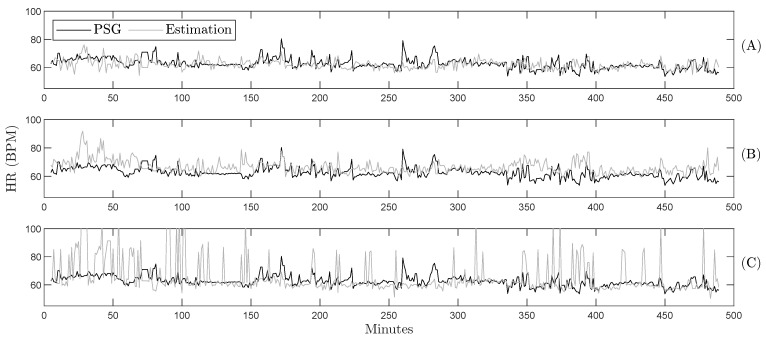
Gold standard and estimated HR during the whole night for one volunteer using the proposed methodology and different estimation techniques: (**A**) zero-crossing, (**B**) peak detection, and (**C**) Burg’s method.

An example of HR prediction throughout the night is presented in Figure 6 for one of the subjects in the database. While cardiac dynamics were challenging to capture using Burg’s method (Figure 6C), zero-crossing (Figure 6A) and peak detection (Figure 6B) effectively represent the increases and decreases in heart rate during the night. For instance, this methodology accurately reflects the HR changes around minute 350 and at the end of the recording when the subject woke up.

A between-subject analysis was also conducted for HR prediction to validate the overall performance of each method. Without distinguishing between frequency bands or sleeping positions, the zero-crossing and peak detection methods stand out, achieving mean accuracies of 89.6% and 88.7%, respectively, whereas Burg’s method reaches a mean accuracy of 85.1%.

**Figure 7:**
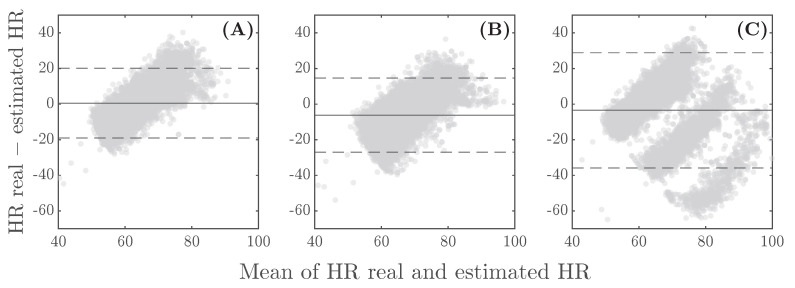
Bland–Altman plots for HR validation using (**A**) zero-crossing, (**B**) peak detection, and (**C**) Burg’s method. Dashed lines represent the limits of agreement (mean difference ± 1.96 standard deviations), and solid lines represent the mean difference between the predicted and measured values.

The overall Bland–Altman plots are shown in Figure 7. Although all methods exhibit small biases of 0.5, −6.2, and −3.5 BPM for zero-crossing, peak detection, and Burg’s method, respectively, the limits of agreement vary significantly. Burg’s method (Figure 7C) shows the widest range, while zero-crossing and peak detection (Figure 7A,B) display relatively narrower limits, between 20 and −20, indicating better agreement for these methods. In both cases, the dispersion increases at lower heart rates, indicating that these methods may have difficulty predicting lower HR values, as suggested by the results in Table 3.

## 5. Discussion

Continuous tracking of heart and respiratory rates throughout the night is crucial for assessing sleep quality and addressing various sleep disorders. For this reason, a robust, contactless and non-invasive methodology, using SB, has been developed to evaluate both variables during sleep, ensuring that minor movements and different sleep positions do not affect the measurements. This is particularly important since data from gyroscopes can easily be contaminated by body movements in this context [19,25]. Nevertheless, contactless detection remains challenging for several reasons. One such challenge is the relative distance between the subject and the sensor, especially for HR detection, as noted by Jia et al. [18]. This is why some systems require the sensor to be placed in very specific areas, typically near the heart [20,26,27] or in locations with a strong pulse, such as the wrist [11,28,29,30,31] or finger [12,32,33]. In SB the IMU sensor is strategically placed at the center of the sheet (see Figure 1). However, as the individual moves away from the center, the heart signal weakens, which may account for some accuracy percentages in Table 3 and the increased dispersion in the Bland–Altman plots for HR detection (Figure 7). A potential strategy to mitigate this limitation is to enhance the hardware configuration by incorporating additional IMU sensors distributed across the sheet, allowing the selection of the sensor closest to the subject’s body for HR prediction and thereby improving signal robustness. In contrast, the strength of the respiratory muscles and the larger area covered by the diaphragm prevent significant signal loss when the subject shifts position. This explains the lower dispersion in the Bland–Altman analysis for RR (Figure 5) and the smaller differences observed in Table 2.

A new pipeline was proposed to estimate heart and respiratory signals from data obtained using a three-axis gyroscope from the SB IMU sensor. Once a clean signal was obtained, with waveforms reflecting cardiac and respiratory patterns, the frequencies were estimated using three different methods. Results demonstrated that the methodology, along with zero-crossing and peak detection techniques, successfully captured the variability in RR and HR, respectively, during the night using the specifications in Table 1. Additionally, these methods are reported to be easily implementable in microcontrollers [33,34], making them suitable for real-time applications.

Recent studies have reported novel, contactless methodologies for calculating RR. In the study of Lee and Yoo [35], RR was estimated using a smartphone accelerometer, with differences of 0, 1, and 2 BrPM compared to the actual data across different subjects. Edanami et al. [14] used radars placed on the torsos of nine subjects, reporting an average difference of 1.1 BrPM from the reference. The Oura ring calculates RR using an ECG-derived method, achieving accuracy within 1 BrPM throughout the entire night [36]. These results are quite similar to those obtained with the proposed zero-crossing method, which yielded a median difference of 0.9 BrPM, with an interquartile range between 0.4 and 1.9 BrPM compared to the PSG reference. Additionally, the method shows no sensitivity to low respiratory frequencies, which are common during rest (as was the case for 80% of the volunteers in this study), and its estimation performance does not depend on sleeping position. The agreement limits in the Bland–Altman total plot (Figure 5) are tight, and the offset is very close to zero, which shows a good agreement between the real RR and the proposed method.

Estimating heart rate based on motion tracking is more challenging than respiratory rate estimation [1]. A study involving 653 volunteers [37] reported an average nighttime HR of 65.9 ± 10.4 BPM, indicating that only a small percentage of the population experiences an HR below 50.0 BPM at night, a range in which the proposed methodology may face limitations (see first row of Table 3). In a high-motion environment, as presented by Thakur et al. [28], the Bland–Altman limits of agreement were 16.6 and −15.2 BPM, similar to those obtained in this study for HR prediction using peak detection. Several recently reported studies aiming to estimate HR using contactless approaches show results comparable to those obtained with the proposed methodology. In the study by Edanami et al. [14], a mean difference of 5.5 BPM is reported, which is comparable to the results of this study for HR values above 55.5 BPM. More generally, other authors, such as Hao et al. [38] and Abbas et al. [39], report differences of up to 10 BPM, similar to those observed in low heart rate ranges when using zero-crossing and Burg’s method. As expected, the power of the cardiac signal reaching the IMU decreases when the person lies in a lateral position on the bed, which is reflected in the increased difference in heartbeats relative to the reference value (see last rows of Table 3). For this reason, studies such as [40] include body position as a variable for HR prediction using machine learning models.

EarSleep [41] is a novel earable-based device that also addresses several technical challenges when estimating HR and RR during sleep, particularly due to the sensor placement and the indirect methods used for signal acquisition, similarly to the approach presented in this study. The authors report mean absolute errors for heartbeat and breathing across all 18 participants ranging from [2.16 to 5.24] BPM and from [1.03 to 3.29] BrPM, respectively, which are comparable to the results obtained with the proposed Smart Bedding system.

As shown in Figure 4A and Figure 6B, the selected methods accurately follow the respiratory and cardiac patterns, respectively. Given that identifying cardiorespiratory interactions during sleep is a powerful tool for assessing sleep quality [42], the SB-derived predictions could therefore be used to obtain sleep quality indices. In this context, SB is intended to complement existing sleep assessment methods by enabling unobtrusive, in-home monitoring, rather than serving as a diagnostic alternative to PSG.

## 6. Conclusions

This study presented a processing methodology for data acquired from a three-axis gyroscope embedded in the novel SB device. It has been demonstrated that cardiorespiratory signals can be accurately obtained, enabling the determination of RR using zero-crossings and HR through peak detection with high precision (overall agreement of 93.9% and 88.7%, respectively) when validated against values derived from a PSG study.

RR detection showed no significant limitations, either across the frequency range or regarding sleeping position. HR estimation exhibited a noticeable decrease in accuracy when HR was low (HR < 55 BPM) and the volunteer was sleeping in a lateral position. However, only a small percentage of the healthy adult population presents such low HR values during sleep. It is suggested to incorporate additional gyroscopes into SB, so that a larger area of the device is covered during sleep and the dependence on body position and cardiac wave power is minimized. This will allow improvements in the detection algorithms.

As with any validation study, increasing the dataset could provide further insights into the robustness of the algorithm and help address the limitations identified.

It is well-established that the use of inertial sensors is highly effective in estimating HR and RR, especially when placed close to the source. This is why their use has become widespread in devices such as rings, watches, and chest straps for applications like sleep monitoring. However, this article demonstrates that with simple signal processing techniques, it is feasible to achieve accurate estimations, promoting the use of these devices in completely non-invasive conditions, even for sleep tracking purposes.

## Figures and Tables

**Table 1:** Temporal and frequency domain techniques used for heart rate (HR) and respiratory rate (RR), specifying the considerations for each method.

Technique	Vital Sign Detection	Specifications	
		RR	HR
Zero-crossing	Number of points at which the signal changes sign divided by 2 × 60	Minimum 1 s between zero-crossing points	Minimum 0.25 s between zero-crossing points
Peak detection	Number of local peaks detected	Minimum 1 s between peaks	Minimum 0.5 s between peaks
Burg’s method	Frequency at the maximum value of the power spectral density estimation × 60	Order: 2	Order: 4

**Table 2:** Respiratory rate estimations and real value comparisons during the whole night using accuracy and differences in BrPM for all volunteers according to sleep position and respiratory rate band. Data are presented as Q2 [Q1–Q3]. *n* represents the number of volunteers or epochs.

Comparison Real vs. Estimation			Diff (BrPM)	Accuracy (%)
Frequency band	12 ≤ RR ≤ 14.4 *n* = 16 volunteers	Zero-crossing	0.8 [0.3–1.5]	94.3 [88.6–97.6]
		Peak detection	2.2 [0.9–3.8]	84.2 [69.9–93.4]
		Burg’s method	1.3 [0.6–2.4]	90.2 [81.7–95.7]
	14.5 ≤ RR ≤ 17.4 *n* = 8 volunteers	Zero-crossing	0.8 [0.3–1.8]	94.6 [88.8–97.9]
		Peak detection	1.0 [0.5–2.0]	93.6 [87.3–97.1]
		Burg’s method	1.0 [0.5–1.8]	93.7 [88.7–97.1]
	17.5 ≤ RR ≤ 20 *n* = 6 volunteers	Zero-crossing	1.7 [0.7–3.3]	90.3 [81.5–96.1]
		Peak detection	1.1 [0.5–2.2]	93.7 [87.7–97.1]
		Burg’s method	1.9 [0.9–3.1]	89.2 [82.9–94.5]
Position in bed	Supine *n* = 7185 epochs	Zero-crossing	0.9 [0.4–1.9]	93.8 [87.3–97.5]
		Peak detection	1.6 [0.7–3.2]	89.0 [76.9–95.4]
		Burg’s method	1.3 [0.6–2.5]	91.0 [83.0–96.0]
	Prone *n* = 90 epochs	Zero-crossing	0.7 [0.4–1.3]	94.8 [90.1–97.3]
		Peak detection	2.5 [1.4–4.0]	82.4 [69.5–90.2]
		Burg’s method	1.0 [0.4–2.3]	93.4 [82.4–96.8]
	Left side *n* = 4540 epochs	Zero-crossing	0.9 [0.4–2.0]	93.7 [87.0–97.3]
		Peak detection	1.6 [0.7–3.1]	89.3 [78.0–95.7]
		Burg’s method	1.4 [0.6–2.4]	90.7 [83.6–95.6]
	Right side *n* = 1837 epochs	Zero-crossing	0.8 [0.3–1.7]	94.7 [89.2–97.9]
		Peak detection	1.0 [0.5–2.1]	93.7 [86.7–97.1]
		Burg’s method	1.2 [0.6–2.1]	92.5 [87.0–96.5]

**Table 3:** Heart rate estimations and real value comparisons during the whole night using accuracy and differences in BPM for all volunteers according to sleep position and heart rate band. Data are presented as Q2 [Q1–Q3]. *n* represents the number of volunteers or epochs.

Comparison Real vs. Estimation			Diff (BPM)	Accuracy (%)
Frequency band	40 ≤ HR ≤ 55.4 *n* = 9 volunteers	Zero-crossing	10.3 [6.7–14.1]	80.0 [71.7–87.7]
		Peak detection	18.0 [13.1–22.3]	66.1 [55.7–76.4]
		Burg’s method	10.3 [5.8–28.4]	79.4 [49.1–89.1]
	55.5 ≤ HR ≤ 70.4 *n* = 15 volunteers	Zero-crossing	4.0 [1.8 - 6.9]	93.9 [89.3–97.2]
		Peak detection	5.3 [2.4–9.5]	92.1 [85.2–96.5]
		Burg’s method	5.7 [2.5–11.9]	91.3 [83.1–96.1]
	70.5 ≤ HR ≤ 100 *n* = 6 volunteers	Zero-crossing	10.0 [6.6–14.0]	86.9 [82.1–91.1]
		Peak detection	3.7 [1.6–7.4]	95.6 [90.6–97.9]
		Burg’s method	11.7 [8.3–15.8]	84.5 [79.6–88.9]
Position in bed	Supine *n* = 7185 epochs	Zero-crossing	6.0 [2.8–10.3]	90.9 [84.3–95.7]
		Peak detection	6.8 [2.9–12.6]	89.8 [79.1–95.9]
		Burg’s method	7.7 [3.4–14.9]	88.3 [78.4–94.6]
	Prone *n* = 90 epochs	Zero-crossing	3.5 [1.5–9.4]	94.4 [82.1–97.6]
		Peak detection	7.4 [2.9–20.7]	88.0 [58.0–95.4]
		Burg’s method	2.2 [1.3–25.1]	96.2 [55.6–97.9]
	Left side *n* = 4540 epochs	Zero-crossing	7.5 [3.5–12.4]	88.2 [79.1–94.6]
		Peak detection	9.8 [3.7–17.2]	84.8 [68.7–94.6]
		Burg’s method	9.3 [4.6–17.4]	85.4 [71.9–92.8]
	Right side *n* = 1837 epochs	Zero-crossing	8.2 [4.1–12.3]	87.2 [80.3–93.9]
		Peak detection	6.6 [2.6–16.0]	90.7 [71.0–96.3]
		Burg’s method	9.4 [5.4–15.1]	85.4 [78.2–91.7]

## Data Availability

The data presented in this study are available on request from the corresponding author.

## References

[1] Zhu K., Li M., Akbarian S., Hafezi M., Yadollahi A., Taati B. (2019). Vision-Based Heart and Respiratory Rate Monitoring During Sleep—A Validation Study for the Population at Risk of Sleep Apnea. IEEE J. Transl. Eng. Health Med..

[2] American Heart Association (2022). Sleep Apnea and Heart Health.

[3] Jean-Louis G., Zizi F., Clark L.T., Brown C.D., McFarlane S.I. (2008). Obstructive sleep apnea and cardiovascular disease: Role of the metabolic syndrome and its components. J. Clin. Sleep Med..

[4] Spira A.P., Chen-Edinboro L.P., Wu M.N., Yaffe K. (2014). Impact of sleep on the risk of cognitive decline and dementia. Curr. Opin. Psychiatry.

[5] Borges C.R., Poyares D., Piovezan R., Nitrini R., Brucki S. (2019). Alzheimer’s disease and sleep disturbances: A review. Arq. Neuro-Psiquiatr..

[6] Claman D., Okeson K., Singer C. (2019). Sleep Disorders. Behavioral Medicine: A Guide for Clinical Practice.

[7] Vulcan R.S., André S., Bruyneel M. (2021). Photoplethysmography in Normal and Pathological Sleep. Sensors.

[8] Alnaggar M., Siam A.I., Handosa M., Medhat T., Rashad M. (2023). Video-based real-time monitoring for heart rate and respiration rate. Expert Syst. Appl..

[9] Vincent J.L., Einav S., Pearse R., Jaber S., Kranke P., Overdyk F.J., Whitaker D.K., Gordo F., Dahan A., Hoeft A. (2018). Improving detection of patient deterioration in the general hospital ward environment. Eur. J. Anaesthesiol..

[10] Adib F., Mao H., Kabelac Z., Katabi D., Miller R.C. (2015). Smart Homes that Monitor Breathing and Heart Rate. CHI ’15: Proceedings of the 33rd Annual ACM Conference on Human Factors in Computing Systems.

[11] Ringeval M., Wagner G., Denford J., Paré G., Kitsiou S. (2020). Fitbit-Based Interventions for Healthy Lifestyle Outcomes: Systematic Review and Meta-Analysis. J. Med. Internet Res..

[12] (2024). Oura Ring. ¿Por Qué Oura? Un Anillo Inteligente Como Ningún Otro. https://ouraring.com/es/why-oura.

[13] Jia Z., Bonde A., Li S., Xu C., Wang J., Zhang Y., Howard R.E., Zhang P. (2017). Monitoring a Person’s Heart Rate and Respiratory Rate on a Shared Bed Using Geophones. SenSys ’17: Proceedings of the 15th ACM Conference on Embedded Network Sensor Systems.

[14] Edanami K., Sun G. (2022). Medical Radar Signal Dataset for Non-Contact Respiration and Heart Rate Measurement. Data Brief.

[15] Yamana Y., Tsukamoto S., Mukai K., Maki H., Ogawa H., Yonezawa Y. (2011). A sensor for monitoring pulse rate, respiration rhythm, and body movement in bed. Annu. Int. Conf. IEEE Eng. Med. Biol. Soc..

[16] Dafna E., Rosenwein T., Tarasiuk A., Zigel Y. (2015). Breathing rate estimation during sleep using audio signal analysis. Annu. Int. Conf. IEEE Eng. Med. Biol. Soc..

[17] Xu Z., Furui A., Jomyo S., Sakagawa T., Morita M., Takai T., Ando M., Tsuji T. (2021). Pressure-based Detection of Heart and Respiratory Rates from Human Body Surface using a Biodegradable Piezoelectric Sensor. Annu. Int. Conf. IEEE Eng. Med. Biol. Soc..

[18] Jia Z., Alaziz M., Chi X., Howard R.E., Zhang Y., Zhang P., Trappe W., Sivasubramaniam A., An N. HB-Phone: A Bed-Mounted Geophone-Based Heartbeat Monitoring System. Proceedings of the 2016 15th ACM/IEEE International Conference on Information Processing in Sensor Networks (IPSN).

[19] Liu G.Z., Guo Y.W., Zhu Q.S., Huang B.Y., Wang L. (2011). Estimation of respiration rate from three-dimensional acceleration data based on body sensor network. Telemed. J. e-Health.

[20] Phan D.H., Bonnet S., Guillemaud R., Castelli E., Thi N.Y.P. Estimation of respiratory waveform and heart rate using an accelerometer. Proceedings of the 2008 30th Annual International Conference of the IEEE Engineering in Medicine and Biology Society.

[21] Sun X., Qiu L., Wu Y., Tang Y., Cao G. (2017). SleepMonitor: Monitoring Respiratory Rate and Body Position During Sleep Using Smartwatch. Proc. ACM Interact. Mob. Wearable Ubiquitous Technol..

[22] Strumpf Z., Gu W., Tsai C.W., Chen P.L., Yeh E., Leung L., Cheung C., Wu I.C., Strohl K.P., Tsai T. (2023). Belun Ring (Belun Sleep System BLS-100): Deep learning-facilitated wearable enables obstructive sleep apnea detection, apnea severity categorization, and sleep stage classification in patients suspected of obstructive sleep apnea. Sleep Health.

[23] Osorio M., Muñoz I.C., Hernández A.M., Castaño F.A., Martinez-Licona F.M., Ballarin V.L., Ibarra-Ramírez E.A., Pérez-Buitrago S.M., Berriere L.R. (2025). Evaluation of Classification Algorithm Performance in Identifying Sleep Positions Using Pressure Signals Recorded with Smart BeddingTM. X Latin American Conference on Biomedical Engineering.

[24] Lin S., Wang L., Huang B., Zhang Y., Wu X., Zhao J. A Pilot Study on BSN-Based Ubiquitous Energy Expenditure Monitoring. Proceedings of the 2009 Sixth International Workshop on Wearable and Implantable Body Sensor Networks.

[25] Nam Y., Kim Y., Lee J. (2016). Sleep Monitoring Based on a Tri-Axial Accelerometer and a Pressure Sensor. Sensors.

[26] Pini N., Ong J.L., Yilmaz G., Chee N.I.Y.N., Siting Z., Awasthi A., Biju S., Kishan K., Patanaik A., Fifer W.P. (2022). An automated heart rate-based algorithm for sleep stage classification: Validation using conventional polysomnography and an innovative wearable electrocardiogram device. Front. Neurosci..

[27] Landreani F., Faini A., Martin-Yebra A., Morri M., Parati G., Caiani E.G. (2019). Assessment of Ultra-Short Heart Variability Indices Derived by Smartphone Accelerometers for Stress Detection. Sensors.

[28] Thakur S., Chao P.C.P., Tsai C.H. (2023). Precision Heart Rate Estimation Using a PPG Sensor Patch Equipped with New Algorithms of Pre-Quality Checking and Hankel Decomposition. Sensors.

[29] Fukushima H., Kawanaka H., Bhuiyan M.S., Oguri K. (2012). Estimating heart rate using wrist-type Photoplethysmography and acceleration sensor while running. Annu. Int. Conf. IEEE Eng. Med. Biol. Soc..

[30] Zhao C., Zeng W., Hu D., Liu H. (2021). Robust Heart Rate Monitoring by a Single Wrist-Worn Accelerometer Based on Signal Decomposition. IEEE Sens. J..

[31] Kim S.W., Choi S.B., An Y.J., Kim B.H., Kim D.W., Yook J.G. (2015). Heart Rate Detection During Sleep Using a Flexible RF Resonator and Injection-Locked PLL Sensor. IEEE Trans. Biomed. Eng..

[32] Babikir S., Abdel-Khair L. (2012). Microcontroller based Heart Rate Monitor using Fingertip Sensors. UofK Eng. J..

[33] Ali A., Desa M.K.M., Ai Ooi C., Al-Gailani S.A., Hafeez M., Abdullah M.N., Zaid M. (2024). Sensorless microcontroller-based zero-crossing detection system for AC signals using a rounding function. Ain Shams Eng. J..

[34] Colak A.M., Manabe T., Shibata Y., Kurokawa F. (2018). Peak Detection Implementation for Real-Time Signal Analysis Based on FPGA. Circuits Syst..

[35] Lee J., Yoo S.K. (2020). Respiration Rate Estimation Based on Independent Component Analysis of Accelerometer Data: Pilot Single-Arm Intervention Study. JMIR mHealth And Uhealth.

[36] Oura Team How Accurate Is Oura’s Respiratory Rate?. https://ouraring.com/blog/how-accurate-is-ouras-respiratory-rate/?_gl=1*1ktp5ln*_ga*ODkyMjM5OTgxLjE3MTEyMjExOTU.*_ga_QY3EB3Q3FY*MTcyNTg5ODk3MC42LjAuMTcyNTg5ODk3MC42MC4wLjA.

[37] Johansen C.D., Olsen R.H., Pedersen L.R., Kumarathurai P., Mouridsen M.R., Binici Z., Intzilakis T., Kober L., Sajadieh A. (2013). Resting, night-time, and 24 h heart rate as markers of cardiovascular risk in middle-aged and elderly men and women with no apparent heart disease. Eur. Heart J..

[38] Hao Z., Gao Y., Tang Y., Wang Y., Fan K., Li F. (2025). FMCW-based contactless heart rate monitoring. Sci. Rep..

[39] Abbas L., Samy S., Ghazal R., Eldeib A.M., ElGohary S.H. Contactless Vital Signs Monitoring for Public Health Welfare. Proceedings of the 2021 9th International Japan-Africa Conference on Electronics, Communications, and Computations (JAC-ECC).

[40] Lin C.L., Sun Z.T., Chen Y.Y. (2023). Air-mattress system for ballistocardiogram-based heart rate and breathing rate estimation. Heliyon.

[41] Han F., Yang P., Feng Y., Jiang W., Zhang Y., Li X.Y. (2024). EarSleep: In-ear Acoustic-based Physical and Physiological Activity Recognition for Sleep Stage Detection. Proc. ACM Interact. Mob. Wearable Ubiquitous Technol..

[42] Grote V., Frühwirth M., Lackner H.K., Goswami N., Köstenberger M., Likar R., Moser M. (2021). Cardiorespiratory Interaction and Autonomic Sleep Quality Improve during Sleep in Beds Made from Pinus cembra (Stone Pine) Solid Wood. Int. J. Environ. Res. Public Health.

